# Fulminant necrotizing fasciitis secondary to *Aeromonas dhakensis* infection: a case report

**DOI:** 10.3389/fmed.2025.1685528

**Published:** 2025-11-05

**Authors:** Xiaoxia Li, Juanxian Gu, Weifang Zhang, Qinhua Zhang, Zebin Fang, Bin Jin, Zhenfei Ou, Xiao Huang

**Affiliations:** ^1^Department of Intensive Care Unit (ICU), Haining People’s Hospital, Jiaxing, China; ^2^Department of Intensive Care Unit (ICU), First Affiliated Hospital, School of Medicine, Zhejiang University, Hangzhou, China; ^3^Department of Neurosurgery, Haining People’s Hospital, Jiaxing, China

**Keywords:** *Aeromonas dhakensis*, necrotizing fasciitis, multiple organ dysfunction syndrome, antibiotic resistance, case report

## Abstract

**Background:**

Necrotizing fasciitis (NF) is a kind of rapidly destructive soft tissue infection with an unfavorable prognosis. It is usually caused by highly virulent bacteria. Recently, the incidence of NF caused by atypical opportunistic pathogens has been increasing. *Aeromonas dhakensis* is increasingly emerging as a new, highly virulent, biofilm-producing, and multidrug resistant cause of soft tissue infection. The current strains of *A. dhakensis* are causing increasingly severe disease, with delayed diagnosis and treatment.

**Case presentation:**

We report a rare case of NF caused by *A. dhakensis* in a 47-year-old man with liver cirrhosis. He presented 2 days after the onset of fever and painful erythema involving his right lower limb. On admission, the patient was febrile and hypotensive with signs of septic shock and necrotizing soft tissue infection. Blood cultures were rapidly positive for *A. dhakensis*. Multiple imaging studies demonstrated diffuse involvement of the soft tissues, and the patient developed multiple organ dysfunction syndrome (MODS). Despite early-onset broad-spectrum antimicrobial therapy, emergency surgical debridement, and organ support, the patient’s condition continued to decline. Given the extremely poor prognosis, the family decided against further treatment and discharged the patient from the hospital.

**Conclusion:**

This case underscores the necessity of not overlooking opportunistic and atypical pathogens, such as *Aeromonas dhakensis*, especially in high-risk susceptible populations, in rapidly progressing soft tissue infections. Early empirical and targeted antimicrobial therapy, prompt surgical intervention, integrated critical care management, and rigorous public water safety measures are all pivotal to improving patient outcomes and preventing similar adverse events.

## Introduction

Necrotizing fasciitis (NF) is a rare but rapidly progressive and highly destructive soft tissue infection that primarily affects the fascia and adjacent structures (1, 2). The condition can lead to extensive tissue necrosis, sepsis, and even multiple organ dysfunction syndrome (MODS) within a short time (1, 2). These infections are associated with high mortality rates, particularly in cases complicated by toxic shock or septic shock (3). NF often presents insidiously in the early stages, with severe pain that is disproportionate to initial skin changes. As the infection rapidly spreads along fascial planes, patients may develop erythema, swelling, tense bullae, and violaceous skin discoloration within hours to days, followed by septic shock and multiorgan failure (3). Early recognition and timely intervention—particularly prompt surgical debridement and empirical antibiotic therapy—are essential to improving patient outcomes (4).

Common causative organisms of NF include Group A *Streptococcus*, *Clostridium perfringens*, *Escherichia coli*, *Staphylococcus aureus*, and *Vibrio vulnificus*, among others (3). High-risk populations typically include individuals with diabetes mellitus, liver cirrhosis, malignancies, and chronic kidney disease—conditions that compromise host immunity (5–8). Recently, driven by the increasing prevalence of comorbidities and widespread antibiotic use, an emerging trend has been observed in which atypical opportunistic pathogens are implicated in NF (3). Among them, *Aeromonas dhakensis*—a non-fermenting Gram-negative bacillus—has gained increasing clinical attention due to its robust biofilm-forming ability and intrinsic resistance to multiple antibiotics, including carbapenems (2, 4). *A. dhakensis* was first isolated in 2002 from cases of diarrhea in children in Dhaka, Bangladesh, and is known to cause zoonotic diseases, particularly in coastal areas (7). It has previously been misidentified as *A. hydrophila*, *A. veronii*, or *A. caviae* by commercial phenotypic tests in clinical laboratories (7, 9). Notably, bacteremia caused by *A. dhakensis* is more lethal than bacteremia caused by other Aeromonas species (7, 9, 10).

Currently, the number of case reports on *A. dhakensis* infections is still quite limited. Existing reports indicate that infections caused by this bacterium can result in a variety of severe conditions. For example, a 26-year-old healthy man died after developing fulminant pneumonia and bacteremia following exposure to river water (11). Another young male succumbed to multiple organ failure caused by *A. dhakensis* infection due to community-acquired pneumonia (12). Two patients with severe dengue fever who were co - infected with this bacterium developed septicemia and necrotizing fasciitis, ultimately leading to death (13). Some patients also presented with empyema, fulminant pneumonia, and bacteremia caused by *A. dhakensis* with poor outcomes (14, 15). Additionally, a case of secondary *A. dhakensis* infection occurring during chemotherapy in a patient with acute T-cell lymphoblastic leukemia, which was successfully treated following adjustment of targeted antibiotic therapy (16).

At present, there are no standardized diagnostic or therapeutic guidelines for infections caused by this bacterium, particularly regarding the integrated management of antimicrobial therapy, surgical intervention, and critical care support following secondary necrotizing fasciitis. Consequently, clinical management strategies are largely informed by case reports and accumulated clinical experience. Herein, we report a rare case of NF caused by *A. dhakensis*, complicated by MODS, in a patient with underlying liver cirrhosis. We detail the clinical course, diagnostic challenges, and therapeutic interventions undertaken. This report may provide valuable reference for future clinical diagnosis and management of similar infections.

## Case description

A 47-year-old male presented to the emergency department at 2:30 a.m. on June 26, 2025, with a 2-day history of fever and painful swelling of the right lower limb. He had a 10-year history of chronic hepatitis B and had been on long-term antiviral therapy with entecavir and ursodeoxycholic acid, with stable disease control. He denied any history of diabetes, trauma, recent surgery, or use of implantable medical devices. It is noteworthy that, despite repeated inquiries by medical staff regarding any recent exposure to seawater or aquatic animals such as fish and shrimp, the patient consistently denied any such contact. On admission, his body temperature was 39.2 °C, heart rate 116 bpm, respiratory rate 26 breaths/min, and blood pressure 88/54 mmHg. He was agitated and irritable. The right lower extremity showed marked swelling, erythema, and severe pain, with elevated local temperature. Dorsalis pedis pulse was diminished, and a small blister was observed in the right popliteal fossa. A diagnosis of soft tissue infection with septic shock was suspected. Blood cultures were collected immediately, and empirical antimicrobial therapy was initiated with meropenem and linezolid.

**FIGURE 1:**
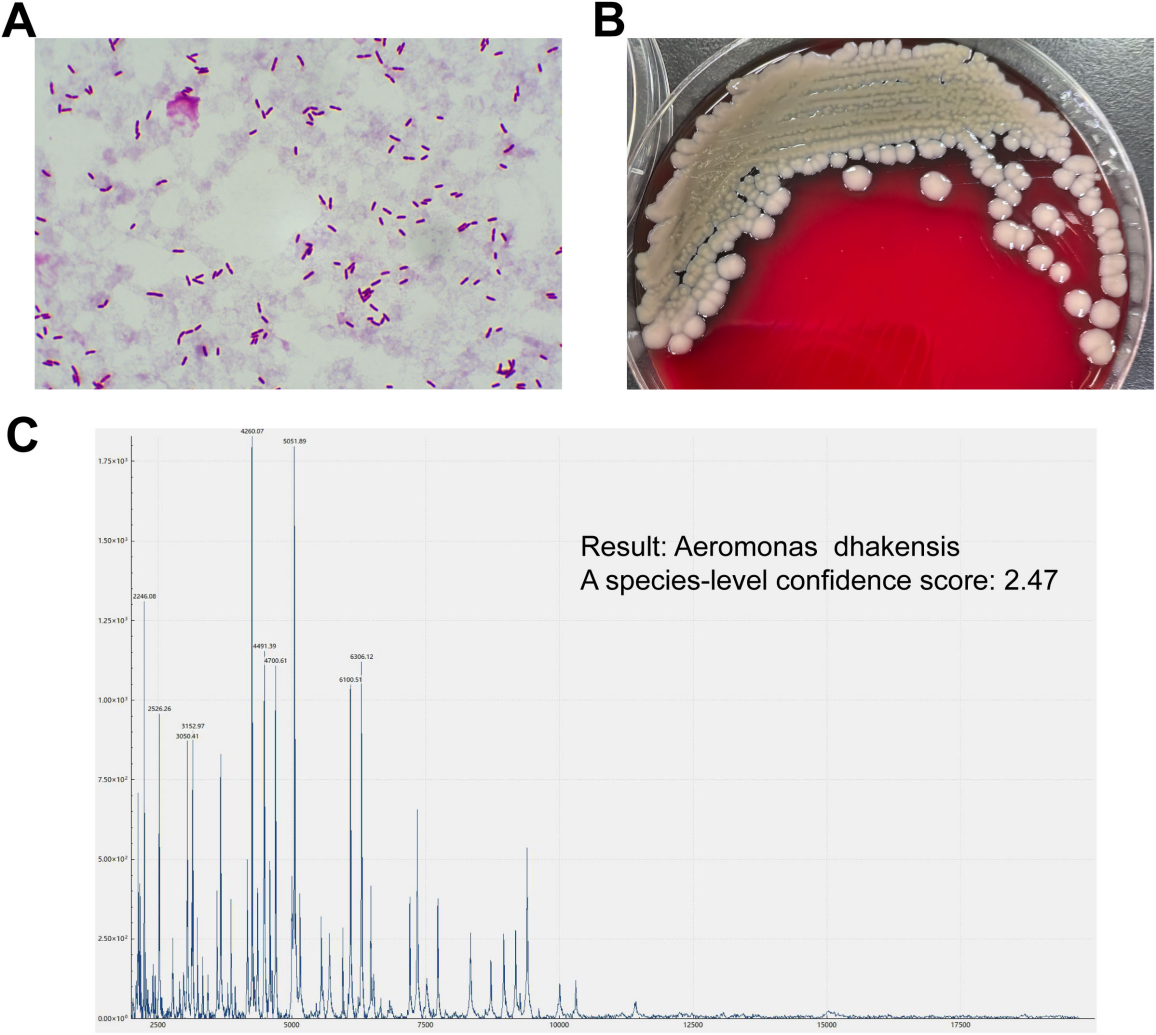
Laboratory-based pathogen identification. **(A)** Gram staining of the blood smear revealed numerous scattered, clearly stained, short gram-negative rods. **(B)** Blood culture plates showed tightly packed, well-defined, smooth and moist, milky-white colonies. **(C)** MALDI-TOF MS analysis identified *Aeromonas dhakensis*, with a score of 2.47, which meets the threshold for confident species-level identification (≥2.0).

**FIGURE 2:**
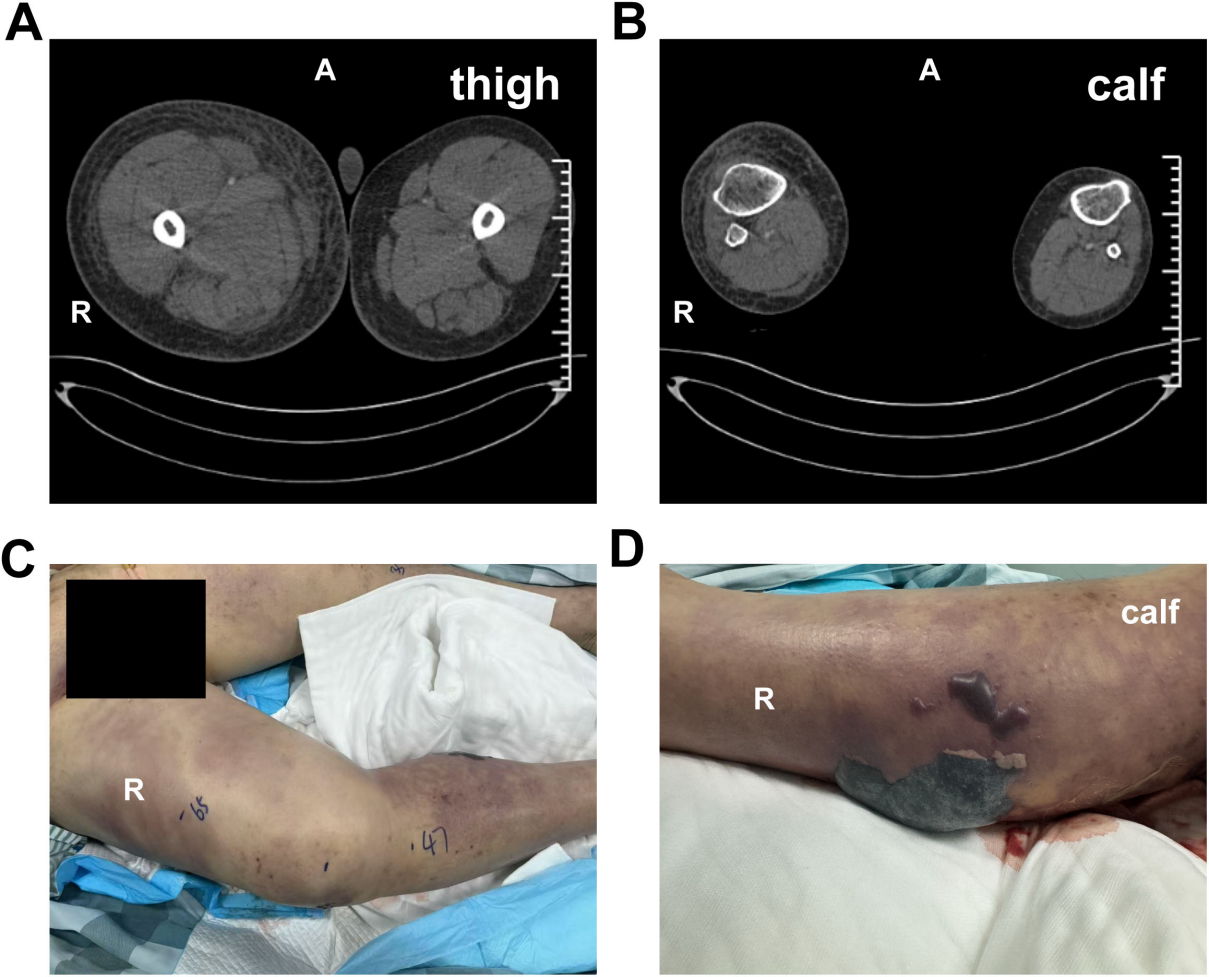
Preoperative imaging and clinical manifestation. Preoperative lower limb CTA revealed extensive subcutaneous fluid accumulation and fascial thickening in the right thigh **(A)** and right lower calf **(B)**, with narrowed yet patent femoral, anterior tibial, and posterior tibial arterial flow tracts. Clinically, the right thigh **(C)** and right lower calf **(D)** showed diffuse erythema and subcutaneous ecchymosis; tense bullae were observed on the posterior aspect of the right lower leg **(D)**.

Approximately 6.5 h later, preliminary blood culture results were positive (Figures 1A, B). Matrix-Assisted Laser Desorption/Ionization Time-of-Flight Mass Spectrometry (MALDI-TOF MS, EXS2000, Zhongyuan Huiji, Beijing, China) identified *A. dhakensis* as the causative organism, with a confidence score of 2.47, indicating reliable species-level identification (Figure 1C). The same pathogen was later confirmed from both aspirated fluid and repeat blood cultures. Laboratory results were consistent with septic shock and multi-organ dysfunction: WBC 6.5 × 10^9^/L, platelets 24 × 10^9^/L, hemoglobin 93 g/L; procalcitonin 28.26 ng/mL, C-reactive protein 52.4 mg/L, interleukin-6 (IL-6) > 5000 pg/mL; arterial blood gas showed pH 7.32, HCO_3_^–^ 14.7 mmol/L, and lactate 19 mmol/L. Markedly elevated liver enzymes were observed (ALT 668 U/L, AST 2824 U/L), along with serum creatinine 260 μmol/L, myoglobin > 3975 ng/mL, D-dimer 3.255 μg/mL, and BNP 2461 pg/mL. Imaging revealed multi-system involvement: echocardiography showed preserved left ventricular ejection fraction (EF 65%) with mild tricuspid regurgitation; abdominal CT indicated cirrhosis, splenomegaly, and mild ascites. CTA of the lower extremity demonstrated extensive subcutaneous edema and fascial thickening in the right lower leg, with narrowed but patent anterior and posterior tibial arteries (Figures 2A, B).

**FIGURE 3:**
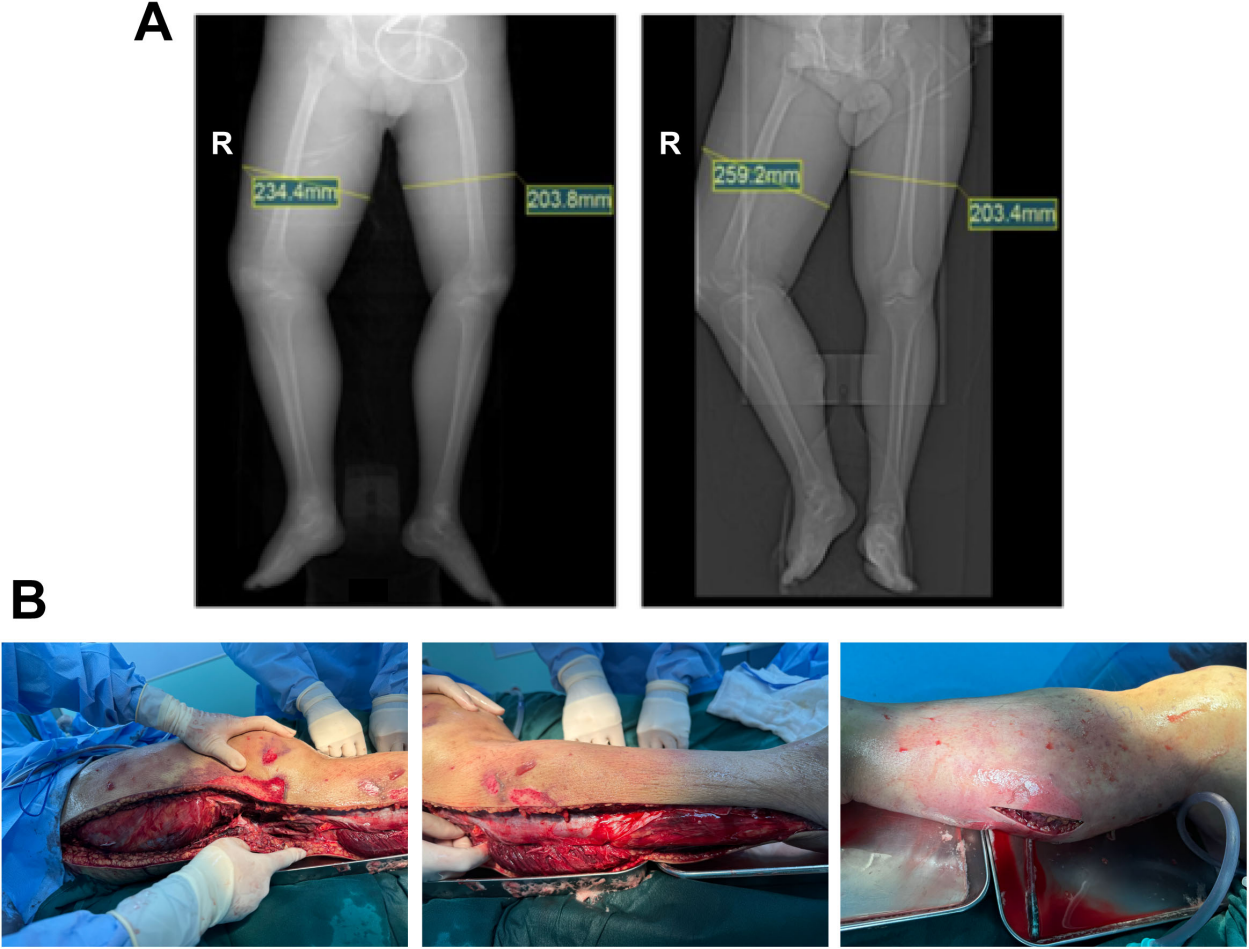
Preoperative imaging and intraoperative findings. (**A**, left) CT localization scan of both lower limbs at 02:17 on June 26, 2025, showed marked thickening of the right thigh soft tissue, measuring 234.4 mm (right thigh) and 203.8 mm (left thigh). (**A**, right) CT scan at 10:26 on June 26, 2025, indicated significant progression of right thigh soft tissue swelling, now measuring 259.2 mm (right thigh) and 203.4 mm (left thigh). **(B)** Intraoperative exploration revealed pronounced edema of the right lower limb fascia and areas of muscle that appeared gray-white and inelastic, consistent with necrotic changes and diagnostic for necrotizing fasciitis.

The patient was immediately managed with fluid resuscitation, mechanical ventilation, empirical antibiotics (later adjusted to meropenem and levofloxacin following pathogen identification), continuous renal replacement therapy (CRRT), vasopressors (norepinephrine and vasopressin), and comprehensive supportive measures including albumin, red blood cells, plasma, and cryoprecipitate transfusions. Progressive tension bullae with hemorrhagic blisters and malodorous exudate rapidly developed in the affected limb (Figures 2C, D). Follow-up CT confirmed worsening soft tissue swelling (Figure 3A). Emergent fasciotomy and decompression were performed by orthopedic surgery on the same day. Intraoperatively, marked fascial edema was observed, with portions of the muscle appearing pale, non-elastic, and exudative—findings consistent with necrotizing fasciitis (Figure 3B). Postoperative management included daily wound care and drainage.

Despite aggressive multiorgan support, the patient’s circulatory and oxygenation status continued to deteriorate, and his condition progressed rapidly. On the evening of June 27, 2025, given the extremely poor prognosis, the family opted to discontinue further treatment and signed a discharge against medical advice. The patient was discharged with ongoing mechanical circulatory and respiratory support.

## Discussion and conclusions

This case involved a 47-year-old male patient with a history of decompensated liver cirrhosis secondary to chronic hepatitis B, representing a high-risk population for NF (8). His immunocompromised status, hypoalbuminemia, coagulopathy, and increased intestinal permeability collectively predisposed him to rapid infection progression and a systemic inflammatory response. Despite atypical local skin manifestations—limited to erythema and blistering—the clinical course deteriorated rapidly into septic shock and MODS. Intraoperative findings of grayish, inelastic muscle, fascial edema, and turbid serosanguinous fluid confirmed the diagnosis of NF. This clinical trajectory underscores the necessity of maintaining a low threshold for surgical intervention, including early fascial exploration and debridement, in highly susceptible hosts—even when imaging or laboratory data are inconclusive. In selected cases, “diagnostic debridement” may serve as a time-critical therapeutic strategy (1–6).

The causative pathogen in this case, *A. dhakensis* (previously misidentified in literature as *A. hydrophila*, *A. caviae*, and *A. veronii*), is a non-fermentative Gram-negative facultative aerobe, ubiquitously found in water sources, moist soil, and hospital environments (7, 17). Clinical infections are predominantly reported in immunocompromised patients, such as those undergoing organ transplantation, receiving chemotherapy, or with indwelling devices (7, 9). While chronic respiratory infections and catheter-related bloodstream infections are more frequently documented, emerging case reports have demonstrated its ability to cause severe soft tissue infections, including NF, particularly in vulnerable hosts where the organism exhibits unexpectedly high virulence and tissue-destructive capacity (13, 18).

*A. dhakensis* exhibits notable multidrug resistance (MDR). Antibiotic selection is a crucial issue that clinicians need to focus on. A previous study found that two biovars of bacteria were isolated from blood cultures before and after treatment with carbapenem antibiotics. The strain isolated at the onset of sepsis was sensitive to carbapenems. However, the strain isolated after treatment was resistant. The expression of the metallo-β-lactamase gene was upregulated in the resistant strain (9). Most clinical isolates of *A. dhakensis* exhibit a high level of resistance to carbapenems, considerably greater than that observed in non-clinical isolates (19). This resistance is primarily associated with the presence of chromosomally encoded metallo-β-lactamase genes, such as *bla*CphA (19). Furthermore, studies have shown that *A. dhakensis* frequently carries the chromosomal AmpC β-lactamase gene *bla*AQU-1. Wu et al. were the first to confirm its presence in clinical isolates and noted that overexpression of this gene can lead to resistance to cefotaxime or induce mutations during treatment (20). Subsequent studies have further confirmed that *bla*AQU-1 has a species - specific distribution in *A. dhakensis* (7, 20, 21). The carriage rate of *bla*AQU-1 in clinical isolates of *A. dhakensis* is generally over 80%–90%, and it is closely associated with resistance to third - generation cephalosporins (20). Although most strains still remain sensitive to cefepime and fluoroquinolones, there are regional and temporal variations in susceptibility. Early studies generally reported a sensitivity rate of over 90% (20), while a recent multi - center study in Japan showed that it had dropped to approximately 80% (22). In contrast, studies in Malaysia and Singapore still reported a sensitivity rate of around 95.7% (19). This trend suggests that even cefepime, a fourth - generation cephalosporin, may face a risk of reduced efficacy in some regions or time periods. In clinical practice, carbapenems are not recommended as monotherapy for serious *A. dhakensis* infections, particularly bloodstream infections; instead, in addition to cefepime and fluoroquinolones, tigecycline has demonstrated good *in vitro* activity against the majority of clinical isolates and possesses favorable tissue penetration, making it a potential treatment option (7, 19, 23). However, since tigecycline exhibits relatively low plasma concentrations and primarily distributes into tissues, its use in patients with concomitant bacteremia should be approached with caution (24, 25).

In our case, both blood cultures and puncture fluid cultures revealed the presence of *A. dhakensis*. The susceptibility results indicated that the strain was sensitive to carbapenems (imipenem and meropenem), fourth-generation cephalosporins (cefepime), fluoroquinolones (levofloxacin), aminoglycosides, and trimethoprim-sulfamethoxazole. Subsequently, the presence of the strain was further confirmed through MetaCAP pathogen nucleic acid high-throughput sequencing, with no resistance genes detected. During the treatment process, due to the initial inability to identify the pathogen, an empirical regimen of imipenem-cilastatin combined with linezolid was used. Once the pathogen was identified, the regimen was adjusted to imipenem-cilastatin combined with levofloxacin. However, the clinical efficacy was suboptimal. This result suggests that infections caused by *A. dhakensis* may lead to treatment failure even in the context of drug susceptibility. Unlike the commonly reported cases of resistance genes such as *bla*AQU-1 and *bla*CphA (20, 21), the strain in this case exhibited a relatively intact susceptibility profile. However, its high virulence and the expression of multiple virulence factors may be key contributors to the poor prognosis (7, 19, 21). *A. dhakensis* is known to carry and express various virulence factors, including motility and biofilm formation mediated by polar and lateral flagella (maf1, lafB) (26), exotoxins such as aerolysin and hemolysin (7, 27), as well as type III and type VI secretion system effector proteins (19, 28, 29). These molecular mechanisms can significantly enhance its invasiveness and lethality, thereby providing a plausible explanation for the clinical outcome in this case. Thus, rapid pathogen identification in conjunction with susceptibility testing is critical, as empirical antibiotic regimens often fail to cover these atypical multidrug-resistant pathogens, resulting in treatment delays (19, 30). Clinicians should also be aware of the potential for induction of carbapenem resistance when treating *A.* infections with carbapenem antibiotics (9).

In this case, MALDI-TOF MS enabled rapid and reliable pathogen identification from blood cultures within 6.5 h (7). Compared with conventional microbiological and biochemical methods, which typically require 48–72 h, MALDI-TOF MS allows for species-level identification within hours by detecting the unique protein mass spectra of microorganisms (31, 32). Its efficiency is particularly advantageous in identifying atypical pathogens, where early microbiological confirmation can directly guide antimicrobial therapy and facilitate the shift from empiric to targeted treatment (33). Several molecular diagnostic techniques, such as multiplex polymerase chain reaction (PCR) and metagenomic next-generation sequencing (mNGS), are continuously being developed and optimized to enhance the identification efficiency of *A. dhakensis* (11, 14, 16, 34, 35). This advancement may enable rapid and precise identification of critical species in clinical settings, significantly improving the diagnostic efficiency and accuracy for infectious diseases in the future.

In managing MODS, the patient received comprehensive supportive care including surgical intervention, targeted antimicrobial therapy, and multiple organ support. Early initiation of continuous renal replacement therapy (CRRT) played a pivotal role in stabilizing both acute kidney injury (AKI) and systemic inflammation (36). CRRT has been shown to effectively remove pro-inflammatory cytokines such as interleukin-6 (IL-6), tumor necrosis factor-α (TNF-α), and interleukin-1β (IL-1β) using high-flux filters, thereby attenuating the cytokine storm and improving hemodynamic stability—particularly when employed early in infection-associated MODS alongside fluid and perfusion management (37, 38). Despite the implementation of broad-spectrum interventions including mechanical ventilation, vasopressor support, and anticoagulation, the patient’s condition deteriorated rapidly, culminating in irreversible MODS and the decision to withdraw care. However, this case highlights the critical importance of the “therapeutic window.” Studies indicate that once NF progresses to MODS, mortality can rise to 70–80%, especially in patients with severe underlying diseases or delays in infection control (6, 8, 33). Notably, there is no difference in mortality rates between amputated and non-amputated patients. The primary treatment method is early and aggressive debridement of the affected skin, subcutaneous fat, and fascia (33).

Therefore, early recognition of high-risk hosts, proactive evaluation of local and systemic disease progression, timely infection source control, and targeted antimicrobial strategies are paramount to improving outcomes (39–41). Although *A. dhakensis* is considered an atypical pathogen, it should not be overlooked in patients with significant comorbidities. Non-fermenting Gram-negative rods may possess pathogenic potential and drug resistance beyond conventional expectations. Facing rapidly evolving infectious diseases, improving clinical vigilance, advancing rapid pathogen diagnostic technologies, fostering multidisciplinary collaboration, and implementing personalized treatment strategies represent key avenues for improving survival in such critically ill patients. From a public health perspective, the detection of carbapenem-resistant strains in aquaculture sources, like edible fish, underscores the need for monitoring water bodies and aquaculture environments to prevent the spread of resistant bacteria to humans through the food chain (19). From the perspectives of pathogenicity and drug resistance mechanisms, the polar (maf1) and lateral (lafB) flagellar genes of *A. dhakensis* participate in biofilm formation through flagella-mediated motility and surface attachment (26). Meanwhile, genomic studies on the resistance mechanisms of *A. dhakensis*, as well as the development of more sensitive antimicrobial susceptibility testing methods, are ongoing (18), and recent research has shown that sanguinarine (SA) possesses significant antibacterial activity against *A. dhakensis* (42). A combined IL-1β targeting strategy may offer a new approach to treating severe soft tissue infections caused by *A. dhakensis* by inhibiting the NLRP3 inflammasome pathway to enhance survival and reduce tissue damage (27). These studies are providing increasingly robust evidence to support the treatment of *A. dhakensis* infection.

## Data Availability

The original contributions presented in this study are included in this article/supplementary material, further inquiries can be directed to the corresponding author.
